# The Association of Khorana Risk Score with Venous Thromboembolism and Overall Survival in Patients with Metastatic Gastric Cancer

**DOI:** 10.3390/medicina61061075

**Published:** 2025-06-11

**Authors:** Ezgi Turkoglu, Goncagul Akdag Topal, Sedat Yıldırım, Oguzcan Kınıkoglu, Nisanur Sarıyar Busery, Tugba Kaya, Hacer Sahika Yıldız, Furkan Turkoglu, Cihad Tatar, Abdullah Sakin, Deniz Isık, Seval Ay Ersoy, Tugba Basoglu, Hatice Odabas, Nedim Turan

**Affiliations:** 1 Department of Medical Oncology, Health Science University, Kartal Dr. Lütfi Kirdar City Hospital, 34865 Istanbul, Turkey; 2 Department of Medical Oncology, Tokat State Hospital, 60100 Tokat, Turkey; 3 Department of General Surgery, Aktif International Hospital, 41275 Kocaeli, Turkey; 4 Private Practice, 34000 Istanbul, Turkey; 5 Department of Medical Oncology, Bahcelievler Medipol Hospital, 34196 Istanbul, Turkey

**Keywords:** Khorana Risk Score, venous thromboembolism, overall survival, metastatic gastric cancer

## Abstract

*Background and Objectives:* Venous thromboembolism (VTE) is a serious complication frequently encountered in cancer patients and is associated with high morbidity. In patients undergoing cancer treatment—particularly those receiving chemotherapy—VTE increases treatment-related complications and has a direct impact on mortality. The development of VTE in oncology patients varies depending on cancer type, treatment protocols, and individual patient characteristics. The Khorana Risk Score (KRS) is a validated risk assessment tool used to estimate the risk of VTE development in patients receiving chemotherapy. KRS provides risk estimations based on the patient’s clinical features, cancer type, and treatment process. This study aims to investigate the prognostic value of the Khorana Risk Score in predicting VTE development and overall survival in patients with metastatic gastric cancer. *Materials and Methods:* This retrospective study used data from 337 metastatic gastric cancer patients who presented to Kartal Dr. Lütfi Kırdar City Hospital between January 2012 and June 2024. Patients were categorized into intermediate- and high-risk groups according to the Khorana Risk Score. The study’s primary endpoints were the development of VTE and overall survival. *Results:* There was no statistically significant difference in VTE incidence (*p* = 0.27) or overall survival (11.9 months vs. 11.5 months, *p* = 0.23) between patients in the intermediate- and high-risk groups. *Conclusions:* These results indicate that the Khorana Risk Score is insufficient in predicting VTE development in patients with metastatic gastric cancer and has a weak association with overall survival outcomes. In conclusion, this study demonstrates the KRS’s inadequacy in predicting VTE and survival outcomes in patients with metastatic gastric cancer, highlighting the need for more tailored approaches.

## 1. Introduction

Gastric cancer ranks fourth among cancer-related causes of death worldwide [1]. It is frequently diagnosed at an advanced, unresectable, or metastatic stage, which significantly contributes to its high mortality rate [2]. These advanced unresectable or metastatic cancers are not curable; thus, the primary goals of systemic therapy are symptom palliation, improvement of quality of life, and prolongation of overall survival.

Cancer patients are at high risk for thrombotic complications due to hypercoagulability. Venous thromboembolism (VTE) is one of the leading causes of morbidity in oncology patients and ranks second among cancer-related causes of death [3,4,5]. The risk of VTE is associated with tumor type, stage, time since diagnosis, patient comorbidities, and the administered cancer treatment [6,7,8]. Tumors of the brain, lung, uterus, bladder, pancreas, stomach, and kidney have the highest one-year incidence rates of VTE. Among individuals with these histological subtypes, those with metastatic disease are 4 to 13 times more likely to develop VTE compared to those with localized disease [9].

The risk of thromboembolic events, particularly in patients receiving systemic treatment, should be assessed at baseline and throughout follow-up using validated risk assessment models [9,10]. Among these, the Khorana Risk Score (KRS) is widely used in ambulatory chemotherapy patients and has been validated across large patient cohorts [6,10]. The KRS is calculated using readily obtainable clinical data in most patients, including primary tumor site, hematological parameters, and body mass index (BMI). Its key advantages are its adaptability to routine clinical practice and lack of additional cost. The use of the Khorana Risk Score is also recommended in the updated 2019 thrombosis guidelines of the American Society of Clinical Oncology (ASCO) [11]. Furthermore, recent studies have shown that a high KRS is not only predictive of thrombosis but also associated with an increased risk of mortality [12,13,14,15]. While this relationship can be partially explained by vascular events (such as pulmonary embolism), it is also believed that clinically silent activation of coagulation plays a role in many patients. It has been shown that oncogenes activate the hemostatic system and that coagulation cascade components interact with tumor cells, facilitating the transition to a metastatic phenotype [16,17].

Studies investigating the relationship between the KRS and mortality have demonstrated that a score of 3 or higher is associated with increased mortality in patients with lung cancer [18]. Additionally, an elevated KRS has been reported to correlate with poor prognosis in metastatic pancreatic cancers [19]. Some prospective studies have also highlighted the score’s ability to predict early death in lung and colorectal cancers [12], [20]. Furthermore, a study indicated that the KRS was independently associated with all-cause mortality within two years in Japanese patients with gastric cancer and colorectal cancer prior to receiving chemotherapy [21].

These results suggest that the Khorana Risk Score (KRS) may serve as a prognostic marker not only for thrombotic risk but also for mortality. Accordingly, we aimed to evaluate the prognostic value of an easily applicable and practical scoring system such as KRS in predicting both the development of venous thromboembolism (VTE) and overall survival in the metastatic subgroup of gastric cancer patients, one of the leading causes of cancer-related death worldwide.

## 2. Materials and Methods

**Figure 1:**
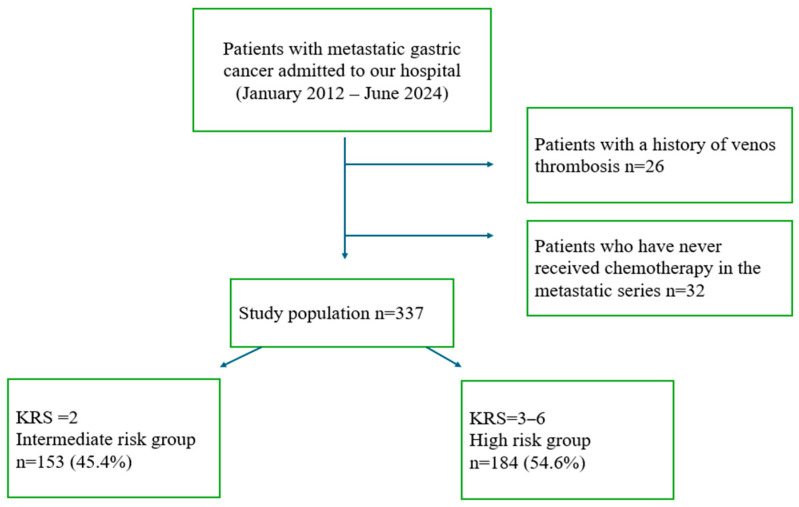
Flowchart depicting the patient selection process. KRS: Khorana Risk Score.

This retrospective study was conducted on patients aged 18 years and older who were diagnosed with gastric cancer and either presented with metastatic disease at diagnosis or developed recurrence/metastases during follow-up, and who received at least one line of systemic treatment. The study included patients admitted to Kartal Dr. Lütfi Kırdar City Hospital between January 2012 and June 2024. Designed as a single-center experience, the study excluded non-metastatic patients who had not received systemic therapy, those with a prior history of deep vein thrombosis (DVT), those under anticoagulant treatment for any reason, or those whose medical records lacked essential data. A total of 337 patients who met the inclusion criteria were included in the final analysis. These patients were stratified into intermediate- and high-risk groups according to the Khorana Risk Score (KRS) (Figure 1).

### 2.1. Data Collection and Study Endpoints

Data of eligible patients were retrospectively retrieved from hospital records. Recorded variables included age, sex, body mass index (BMI), hemoglobin, leukocyte, and platelet counts, tumor histological subtype and grade, metastatic status at diagnosis and sites of metastasis, Khorana score, occurrence of VTE, treatment regimens received, and overall survival data.

In our study, histologic subtypes were categorized according to the World Health Organization (WHO) classification [22]. At the time of diagnosis, metastatic disease was confirmed using computed tomography or positron emission tomography imaging. The presence of an “omental cake” on imaging was considered indicative of omental metastasis and was recorded as such. For first-line treatment, chemotherapy regimens were selected based on patients Eastern Cooperative Oncology Group (ECOG) performance status and overall treatment suitability, as assessed by the treating physician. Patients with ECOG performance status scores of 0 or 1 received 5-fluorouracil (5-FU)-based chemotherapy regimens that included either platinum or irinotecan agents. Patients with an ECOG performance status of 2–3 received single-agent chemotherapy regimens based on the treating physician’s preference. A VTE event was defined as a composite of radiologically confirmed symptomatic or incidental distal or proximal lower-extremity deep vein thrombosis, upper-extremity deep vein thrombosis, or pulmonary embolism occurring between the initiation of anticancer treatment and either completion of the treatment line or death, whichever occurred first. All VTE cases were confirmed by evaluating clinical records and radiographic reports. The follow-up period for VTE assessment lasted until the time of death.

The Khorana score was calculated by assigning points based on tumor site, hematologic parameters, and BMI (Table 1). According to this scoring system, patients were categorized into low (0 points), intermediate (1–2 points), and high (3–6 points) risk groups. As gastric cancer automatically confers at least 2 points in the scoring system, there were no patients in our cohort with a score of 0 or 1. Therefore, patients were divided into two groups: intermediate- and high-risk.

Overall survival (OS) was defined as the time from diagnosis of metastatic disease to death. The primary endpoints of the study were OS and VTE occurrence.

### 2.2. Statistical Analysis

Data were analyzed using SPSS version 22.0 (SPSS Inc., Chicago, IL, USA). Categorical variables were compared using Chi-square and Fisher’s exact tests. For comparisons of numerical variables between two independent groups, Student’s t-test was applied for normally distributed data, while the Mann–Whitney U test was used for non-normally distributed data. Multivariable survival analysis was performed using the Cox proportional hazards regression model. Both univariable and multivariable Cox regression models were constructed, and hazard ratios (HRs) with 95% confidence intervals (CIs) were reported. Variables with a *p*-value less than 0.10 in univariable analysis were considered for inclusion in the multivariable model.

## 3. Results

### 3.1. Clinicopathological Characteristics in the Overall Study Population

**Figure 2:**
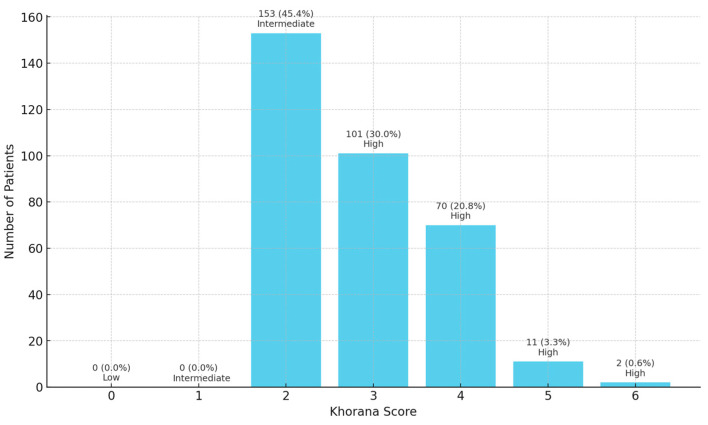
Patient distrubution by Khorana Score.

The baseline demographic and clinicopathological characteristics of the study population are summarized in Table 2. According to the Khorana Risk Score, 45.4% of patients (*n* = 153) were classified as intermediate risk, while 55.6% (*n* = 184) were in the high-risk group (Figure 1). The number and percentage of patients categorized by Khorana Score (0–6) are shown in Figure 2.

The median age at diagnosis was 62 years (range: 21 to 90 years). ECOG performance status was 0–1 in 89.2% of patients (*n* = 302) and 2–3 in 10.4% (*n* = 35). The cohort comprised 105 female (31.2%) and 232 male (68.8%) patients. A total of 115 patients (34.2%) had a history of surgical intervention for the primary gastric tumor. Regarding tumor histology, 255 patients (75.9%) had adenocarcinoma, 17 (5.1%) had mucinous adenocarcinoma, and 64 (19.0%) had signet ring cell carcinoma. Regarding tumor differentiation, 80 patients (34.5%) had well to moderately differentiated tumors, whereas 152 patients (65.5%) had poorly differentiated or undifferentiated tumors. HER2 (Human Epidermal Growth Factor Receptor 2) positivity was detected in 74 patients (24.6%), with 58 (78.4%) receiving anti-HER2 therapy. Mismatch repair (MMR) status was known for 110 patients, all classified as proficient MMR (p-MMR). At the time of diagnosis, 257 patients (76.3%) presented with metastatic disease. A total of 143 patients (42.4%) received FOLFOX (Oxaliplatin plus leucovorin and short-term infusional fluorouracil) or XELOX (Capecitabine and oxaliplatin) regimens, 23 patients (6.8%) were treated with FOLFIRI (Irinotecan plus leucovorin and short-term infusional fluorouracil), 61 patients (18.1%) received DCF (Docetaxel, cisplatin plus leucovorin, and long term infusional fluorouracil), 45 patients (13.4%) were administered cisplatin plus 5-FU (Cisplatin and long term infusional fluorouracil), and 15 patients (4.5%) underwent the FLOT (Oxaliplatin, leucovorin plus docetaxel, and short-term infusional fluorouracil) regimen.

### 3.2. Clinicopathological Characteristics Intermediate and High Khorana Scores

There were no statistically significant differences between the intermediate- and high-risk groups regarding age at diagnosis, sex, ECOG performance status, prior surgery for the primary tumor, tumor grade, HER2 (Human epidermal growth factor receptor 2) status, or MMR status. No differences were observed between the groups in the distribution of adenocarcinoma and mucinous adenocarcinoma. However, signet ring cell carcinoma incidence was significantly higher in the intermediate-risk group than in the high-risk group (24% vs. 14.7%, *p* = 0.019). Among the intermediate-risk group, 34 patients (30.1%) had metastatic disease at diagnosis, compared to 25 patients (23.4%) in the high-risk group, a statistically significant difference (*p* = 0.029). No significant differences were found between the two groups regarding the chemotherapy regimens administered in the first-line metastatic setting (*p* = 0.89). When metastasis sites were examined, there were no significant differences between the groups concerning ovary, bone, peritoneal, lung, or peritoneal metastases. However, liver metastasis was significantly more common in the high-risk group (56.0%) compared to the intermediate-risk group (41.2%) (*p* = 0.009).

#### 3.2.1. Khorana Score and VTE Prediction

VTE occurred in 4.6% of patients (*n* = 7) in the intermediate-risk group and 7.6% (*n* = 14) in the high-risk group. However, this difference was not statistically significant (*p* = 0.27).

#### 3.2.2. Khorana Score and Overall Survival

**Figure 3:**
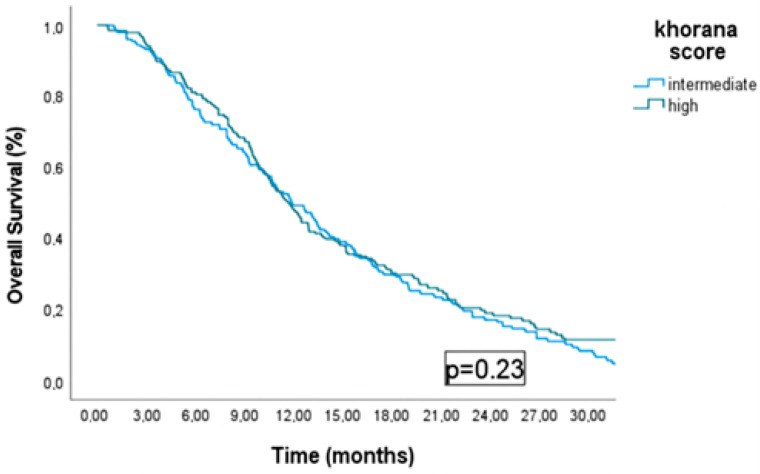
Overal survival in intermediate and high risk groups.

As shown in Figure 3, no statistically significant difference in overall survival was observed between the intermediate- and high-risk groups (11.9 months vs. 11.5 months, *p* = 0.23).

#### 3.2.3. Association Between Clinicopathological Factors and Overall SurvivalfF.T

We performed univariable and multivariable Cox proportional hazards regression to identify factors associated with overall survival (Table 3). In the analysis of demographic and baseline characteristics, age was not significantly associated with overall survival (univariable HR: 1.01 per year; 95% CI: 0.99–1.02; *p* = 0.286; multivariable HR: 1.00; 95% CI: 0.99–1.02; *p* = 0.658). Similarly, sex (female vs. male) did not have a significant effect (univariable HR: 0.87; 95% CI: 0.68–1.12; *p* = 0.285; multivariable HR: 0.90; 95% CI: 0.68–1.19; *p* = 0.476). No statistically significant difference in survival was observed between the risk groups defined by the Khorana Risk Score (intermediate vs. high risk: univariable HR: 1.16; 95% CI: 0.91–1.46; *p* = 0.230; multivariable HR: 1.11; 95% CI: 0.86–1.43; *p* = 0.429).

In the parameters of performance status and de novo metastatic presentation, patients with an ECOG performance status of 2–3 had a 52% higher risk of death in the univariable analysis (HR: 1.52; 95% CI: 1.06–2.20; *p* = 0.024); however, this association weakened and lost statistical significance after multivariable adjustment (HR: 1.41; 95% CI: 0.94–2.13; *p* = 0.098). Similarly, patients who were not de novo metastatic demonstrated a non-significant trend toward worse overall survival compared to those with de novo metastatic disease (univariable HR: 1.27; 95% CI: 0.96–1.68; *p* = 0.090; multivariable HR: 1.28; 95% CI: 0.95–1.72; *p* = 0.108).

The mucinous and signet-ring cell histologic subtypes also failed to reach statistical significance after multivariable analysis (mucinous adenocarcinoma: HR 1.25; 95% CI 0.65–2.40; *p* = 0.502; signet-ring cell adenocarcinoma: HR 0.74; 95% CI 0.47–1.18; *p* = 0.209). Similarly, the presence of a signet-ring cell component was not found to be significant (HR 1.13; 95% CI 0.76–1.68; *p* = 0.557).

While liver (multivariable HR 0.81; 95% CI 0.61–1.06; *p* = 0.128), lung (HR 1.11; 95% CI 0.81–1.51; *p* = 0.522), bone (HR 1.09; 95% CI 0.75–1.60; *p* = 0.643), and peritoneal metastasis (HR 1.05; 95% CI 0.77–1.43; *p* = 0.772) showed no independent prognostic impact, omental metastasis emerged as a robust adverse factor. Patients with omental involvement had a 70% higher hazard in univariable analysis (HR 1.70; 95% CI 1.22–2.36; *p* = 0.002) and a 68% higher hazard after adjustment for all other covariates (multivariable HR 1.68; 95% CI 1.12–2.53; *p* = 0.013).

## 4. Discussion

Identifying a clinically accessible and reliable predictor of overall survival in patients with metastatic gastric cancer is of great importance for improving patient care. In our study, no statistically significant difference in overall survival was observed between intermediate- and high-risk groups as classified by the KRS. Furthermore, the KRS-based stratification failed to predict early thromboembolic events effectively.

In our cohort, 55.6% of patients were classified as high-risk and 45.4% as intermediate-risk, according to KRS. However, these two groups had no statistically significant difference in VTE incidence. In the literature, a prospective study involving only lung cancer patients reported that a high KRS was ineffective in predicting thrombotic events [23]. Likewise, in a study involving patients with uterine neoplasms, no significant difference in KRS was found between those who developed VTE and those who did not [24]. In a cohort study of patients with pancreatic cancer—a malignancy, like gastric cancer, considered high-risk for VTE—the KRS was found inadequate in distinguishing the likelihood of VTE between intermediate- and high-risk groups [25]. Another study on pancreatic cancer patients found no difference in baseline KRS between those who developed VTE and those who did not [26].

The Khorana Score is a validated tool for predicting chemotherapy-associated VTE in cancer patients. It was derived from a cohort of 2701 patients and validated in an independent cohort of 1365 patients. While its negative predictive value was notably high (98.5%) in the validation cohort, its positive predictive value was less than 7% [9]. Importantly, only 1.4% of patients in that study had gastric or pancreatic cancer, and approximately 35% had metastatic disease. In contrast, all patients in our study had metastatic gastric cancer, representing a more homogeneous population. This difference may explain our findings’ absence of a significant association between KRS and VTE.

A study involving 235,149 cancer cases from U.S. cancer registries identified metastatic disease at diagnosis as a strong predictor of VTE [27]. Although we did not observe a difference in VTE incidence between KRS-defined groups, a higher frequency of VTE would typically be expected in the high-risk group. In our study, de novo metastatic disease was more common in the intermediate-risk group, which may explain the lack of difference in DVT rates between groups.

It is well established that the incidence of venous thromboembolism (VTE) varies across different ethnic groups [27,28]. Various factors, such as comorbidities, the use of central venous catheters, metastatic disease status, and differences in chemotherapy regimens, can influence VTE incidence. We believe that these factors may have contributed to the lack of a statistically significant difference in VTE incidence between the intermediate- and high-risk groups in our study.

In our study, the signet ring cell histologic subtype was not found to have a statistically significant impact on overall survival in the multivariable analysis (signet ring cell adenocarcinoma: HR: 0.74; 95% CI: 0.47–1.18; *p* = 0.209). Similarly, in a large-scale study by Taghavi et al. involving 10,246 patients with gastric adenocarcinoma, no significant difference in prognosis was observed between signet ring cell and non-signet ring cell subtypes when patients were compared at the same disease stage [29]. In our study, omental metastasis was identified as a significant adverse prognostic factor, associated with a 70% increase in the risk of death. Supporting this finding, a previous study demonstrated that cytokines and exosomes secreted from omental tissue promote proliferation, invasion, chemoresistance, and angiogenesis in gastric cancer cells, thereby contributing to poor prognosis [30,31]. A study in the literature reported that omental metastasis was associated with decreased overall survival and may serve as a prognostic marker for disease recurrence and survival [32]. These findings are consistent with and reinforce the prognostic implications observed in our analysis. Liver metastasis was not identified as an independent prognostic factor for overall survival in the multivariate analysis. Although conflicting results have been reported in the literature, the study by Zhang et al. supports our findings [33]. On the other hand, a study reported higher overall mortality in patients with liver metastasis [34]. This finding may be explained by the fact that the study included only patients with a single metastatic site, specifically those presenting with isolated liver metastasis.

In our study, the median OS was 11.9 months in the intermediate-risk group and 11.5 months in the high-risk group. These findings are consistent with previously reported survival outcomes in patients with metastatic gastric cancer [35,36]. In the phase III ToGA trial conducted in patients with HER2-positive gastric and gastroesophageal junction tumors, the addition of trastuzumab significantly improved overall survival (median 14 vs. 11 months; hazard ratio (HR): 0.74, 95% CI: 0.6–0.91) [37]. Approximately 25% of our cohort was HER2-positive, and among these patients, around 78% received trastuzumab therapy. In recent years, the integration of immunotherapy and targeted agents has led to promising improvements in overall survival. For example, in a subgroup analysis involving 44 patients with dMMR/MSI-H (deficient-Mismatch Repair/Microsatellite Instability-High) tumors, the addition of nivolumab to chemotherapy significantly improved OS (HR: 0.34, 95% CI: 0.16–0.74) [38]. Similarly, studies conducted in advanced-stage dMMR/MSI-H gastric adenocarcinomas have demonstrated that the addition of pembrolizumab to chemotherapy has a favorable impact on survival outcomes [39,40]. In our study, all patients whose MMR status was assessed were found to have proficient MMR and were treated with conventional chemotherapy regimens.

A large Japanese cohort study of 27,687 cancer patients identified a KRS cut-off of 2 as predictive of mortality, and the authors recommended classifying risk as low (KRS < 2) or intermediate–high (KRS ≥ 2) for all-cause mortality prediction [41]. In our study, the lack of a significant difference in mortality between the intermediate- and high-risk groups may be attributed to the fact that all patients had a KRS ≥2. Another study demonstrated that the KRS was independently associated with 2-year all-cause mortality in patients with gastric and colorectal cancers [21]. In a prospective study by Sohal et al., the KRS was shown to predict 6-month mortality in colorectal cancer patients receiving chemotherapy [20]. Similarly, Shiba et al. reported significantly lower overall survival in the high-risk KRS group compared to the intermediate- and low-risk groups among gastrointestinal cancer patients receiving chemotherapy (*p* = 0.002) [42]. However, in that study, gastric cancer patients accounted for only 20.8% of the cohort, whereas in our study, all patients had metastatic gastric cancer. This difference in patient composition may explain the lack of a significant difference in overall survival between our groups.

### Limitations

Given the study’s retrospective design, detailed information on systemic treatments, treatment-related toxicities, mortality causes, and some missing data could not be fully retrieved. Differences in clinical practice and how outcomes were recorded may have introduced heterogeneity, potentially affecting results. Treatment-related factors that may influence overall survival—such as treatment response, chemotherapy-related toxicities, the number of administered cycles, and treatment completion rates—as well as underlying conditions and comorbidities that could increase the risk of VTE, were not evaluated in this study. In addition, MMR status could not be determined for all patients. Due to healthcare coverage and reimbursement limitations in Türkiye, patients did not have access to immunotherapy, and only a limited proportion were able to receive anti-HER2 targeted therapy. This study was designed as a retrospective observational cohort consisting entirely of Turkish patients with metastatic gastric tumors. Therefore, the generalizability and external validity of the findings are limited. While the data were obtained from a single center, the large number of included patients helped to minimize selection bias.

## 5. Conclusions

In this study, we concluded that the Khorana Risk Score (KRS) is inadequate in predicting VTE and overall survival outcomes in patients with metastatic gastric cancer. This finding underscores the limitations of KRS in this specific patient population and suggests the need for more tailored risk assessment models that consider the unique clinical characteristics of gastric cancer patients. Several factors may have influenced our findings, including underlying conditions that affect deep vein thrombosis, ethnic background, the metastatic status of the disease, and variations in chemotherapy regimens. Additionally, missing data regarding chemotherapy, such as the number of treatment cycles, adverse effects, treatment response, and cycle completion rates, may have impacted our findings. Future research should focus on developing and validating more specific risk assessment tools that incorporate these variables to enhance the predictive accuracy for VTE and survival in metastatic gastric cancer patients. Furthermore, exploring the clinical application of these findings could lead to improved patient management strategies, potentially reducing morbidity and mortality in this high-risk population.

## Figures and Tables

**Table 1:** Khorana Risk Score.

Risk Factors	Points
Site of primary tumor	
Very high risk (stomach, pancreas)	2
High risk (lung, lymphoma, gynecologic, bladder, testicular)	1
All other sites	0
Pre-chemotherapy platelet count ≥ 350,000/microL	1
Hemoglobin level < 10 g/dL or use of ESAs	1
Pre-chemotherapy WBC > 11,000/microL	1
BMI ≥ 35 kg/m^2^	1

WBC: White Blood Cell, BMI: Body Mass Index.

**Table 2:** Data on Pathological and Clinical Characteristics.

			Khorana Score		
		Total (*n* = 337)	Intermediate (*n* = 153)	High (*n* = 184)	*p* Value
Diagnosis age (year)	Median (min-max)	62.0 (21–90)	62 (23–90)	62 (21–83)	0.990
Gender	Female	105 (31.2)	44 (28.8)	61 (33.2)	0.410
	Male	232 (68.8)	109 (71.2)	123 (68.8)	
ECOG	0–1	302 (89.6)	138 (90.2)	164 (89.1)	0.179
	2–3	35 (10.4)	15 (9.8)	20 (10.9)	
Primary tumor surgery (*n* = 336)	Present	115 (34.2)	61 (40.1)	54 (29.3)	0.490
	Absent	221 (65.8)	91 (59.9)	130 (70.7)	
Histological type of tumor (*n* = 336)	Adenocarcinoma and other subtypes	255 (75.9)	108 (71.0)	147 (79.9)	0.400
	Adenocarcinoma, mucinous	17 (5.1)	7 (5.0)	10 (5.4)	0.422
	Adenocarcinoma, signet-ring cell	64 (19.0)	37 (24.0)	27 (14.7)	***0.019***
Grade (*n* = 232)	Well-moderately differentiated	80 (34.5)	42 (36.8)	38 (32.2)	
	Poorly- Undifferentiated	152 (65.5)	72 (63.2)	80 (67.8)	0.491
Signet ring cell component	Present	108 (32.0)	59 (38.6)	49 (26.6)	***0.026***
	Absent	229 (68.0)	94 (61.4)	135 (73.4)	
HER2 status (*n* = 301)	positive	74 (24.6)	33 (24.6)	41 (24.6)	1.000
	negative	227 (75.4)	101 (75.4)	126 (75.4)	
Herceptin treatment (*n* = 74)	Yes	58 (78.4)	26 (78.8)	32 (78.0)	0.100
	No	16 (21.6)	7 (21.2)	9 (22.0)	
Denova metastatic	Yes	257 (76.3)	34 (30.1)	25 (23.4)	***0.029***
	No	80 (23.7)	79 (69.9)	82 (76.6)	
First line CT regimen					0.895
	FOLFOX/XELOX	143 (42.4)	62 (40.5)	81 (44.0)	
	FLOT	15 (4.5)	8 (5.2)	7 (3.8)	
	FOLFIRI	23 (6.8)	12 (7.8)	11 (6.0)	
	DCF	61 (18.1)	30 (19.6)	31 (16.8)	
	CIS FU	45 (13.4)	20 (13.1)	25 (13.6)	
	Others	50 (14.8)	21 (13.7)	29 (15.8)	
Ovarian metastasis	Present	15 (4.5)	5 (3.3)	10 (5.4)	0.430
	Absent	322 (95.5)	148 (96.7)	174 (94.6)	
Bone metastasis	Present	41 (12.2)	21 (13.7)	20 (10.9)	0.504
	Absent	296 (87.8)	132 (86.3)	164 (89.1)	
Lung metastasis	Present	65 (19.3)	34 (22.2)	31 (16.8)	0.216
	Absent	272 (80.7)	119 (77.8)	153 (83.2)	
Peritoneal metastasis	Present	113 (33.5)	52 (34)	61 (33.2)	0.908
	Absent	224 (66.5)	101 (66)	123 (66.5)	
Omental metastasis	Present	47 (13.9)	23 (13)	24 (15)	0.638
	Absent	290 (86.1)	130 (85)	160 (87)	
Liver metastasis	Present	166 (49.3)	63 (41.2)	103 (56.0)	***0.009***
	Absent	171 (50.7)	90 (58.8)	81 (44.0)	

ECOG: Eastern Cooperative Oncology Group, HER2: Human epidermal growth factor receptor 2, CT: Chemotherapy, FOLFOX: Oxaliplatin plus leucovorin and short-term infusional fluorouracil, FOLFIRI: Irinotecan plus leucovorin and short-term infusional fluorouracil, XELOX: Capecitabine and oxaliplatin, FLOT: Oxaliplatin, leucovorin plus docetaxel, and short-term infusional fluorouracil, DCF: Docetaxel, cisplatin plus leucovorin, and long term infusional fluorouracil CIS FU: Cisplatin and long term infusional fluorouracil. The *p*-values indicating statistical significance were written in bold and italics for emphasis.

**Table 3:** Cox Regression Analyses for Overall Survival in Patients with Metastatic Gastric Cancer.

Dependent		All	HR (Univariable)	HR (Multivariable)
Age	Mean (SD)	61.1 (11.7)	1.01 (0.99–1.02, *p* = 0.286)	1.00 (0.99–1.02, *p* = 0.658)
Khorana Risk Score	High	184 (54.6)	-	-
	Intermediate	153 (45.4)	1.16 (0.91–1.46, *p* = 0.230)	1.11 (0.86–1.43, *p* = 0.429)
Gender	Female	105 (31.2)	-	-
	Male	232 (68.8)	0.87 (0.68–1.12, *p* = 0.285)	0.90 (0.68–1.19, *p* = 0.476)
ECOG	0–1	302 (89.6)	-	-
	1–2	35 (10.4)	1.52 (1.06–2.20, ***p = 0.024***)	1.41 (0.94–2.13, *p* = 0.098)
Histological type of tumor (*n* = 336)	Adenocarcinoma and other subtypes	255 (76.0)	-	-
	Adenocarcinoma, mucinous	17 (5.0)	1.66 (0.92–2.98, *p* = 0.092)	1.25 (0.65–2.40, *p* = 0.502)
	Adenocarcinoma, signet-ring cell	64 (19.0)	1.01 (0.76–1.34, *p* = 0.954)	0.74 (0.47–1.18, *p* = 0.209)
Stone ring component	Absent	229 (68.0)	-	-
	Present	108 (32.0)	1.11 (0.87–1.42, *p* = 0.409)	1.13 (0.76–1.68, *p* = 0.557)
De novo metastatic	Yes	257 (76.3)	-	-
	No	80 (23.7)	1.27 (0.96–1.68, *p* = 0.090)	1.28 (0.95–1.72, *p* = 0.108)
Liver Metastasis	Absent	171 (50.7)	-	-
	Present	166 (49.3)	0.81 (0.64–1.02, *p* = 0.070)	0.81 (0.61–1.06, *p* = 0.128)
Peritoneal Metastasis	Absent	224 (66.5)	-	-
	Present	113 (33.5)	1.26 (0.99–1.61, *p* = 0.065)	1.05 (0.77–1.43, *p* = 0.772)
Omental Metastasis	Absent	290 (86.1)	-	-
	Present	47 (13.9)	1.70 (1.22–2.36, ***p = 0.002***)	1.68 (1.12–2.53, ***p = 0.013***)
Lung Metastasis	Absent	272 (80.7)	-	-
	Present	65 (19.3)	1.04 (0.78–1.39, *p* = 0.778)	1.11 (0.81–1.51, *p* = 0.522)
Over Metastasis	Absent	322 (95.5)	-	-
	Present	15 (4.5)	0.98 (0.56–1.72, *p* = 0.957)	0.85 (0.45–1.59, *p* = 0.610)
Bone Metastasis	Absent	296 (87.8)	-	-
	Present	41 (12.2)	1.05 (0.74–1.48, *p* = 0.802)	1.09 (0.75–1.60, *p* = 0.643)

The statistically significant values have been written in bold and italics for emphasis.

## Data Availability

The data presented in this study are available on request from the corresponding author.

## Abbreviations

The following abbreviations are used in this manuscript:

VTE	Venous thromboembolism
KRS	The Khorana Risk Score
DVT	Deep vein thrombosis
PE	Pulmonary embolism
BMI	Body Mass Index
OS	Overall survival
MMR	Mismatch repair
pMMR	Proficient mismatch repair
ECOG	Eastern Cooperative Oncology Group
HER2	Human epidermal growth factor receptor 2
CT	Chemotherapy
FOLFOX	Oxaliplatin plus leucovorin and short-term infusional fluorouracil
FOLFIRI	Irinotecan plus leucovorin and short-term infusional fluorouracil
XELOX	Capecitabine and oxaliplatin
FLOT	Oxaliplatin, leucovorin plus docetaxel and short-term infusional fluorouracil
DCF	Docetaxel, cisplatin plus leucovorin and long term infusional fluorouracil
CIS FU	Cisplatin and long term infusional fluorouracil
HR	Hazard ratio
CI	Confidence interval
